# NKX3-2 Induces Ovarian Cancer Cell Migration by HDAC6-Mediated Repositioning of Lysosomes and Inhibition of Autophagy

**DOI:** 10.3390/cells13211816

**Published:** 2024-11-04

**Authors:** Alessandra Ferraresi, Ian Ghezzi, Amreen Salwa, Andrea Esposito, Danny N. Dhanasekaran, Ciro Isidoro

**Affiliations:** 1 Laboratory of Molecular Pathology, Department of Health Sciences, Università del Piemonte Orientale “A. Avogadro”, Via Solaroli 17, 28100 Novara, Italy; 20020210@studenti.uniupo.it (I.G.); salwa.amreen@uniupo.it (A.S.); andrea.esposito@uniupo.it (A.E.); 2 Stephenson Cancer Center, The University of Oklahoma Health Sciences Center, Oklahoma City, OK 73104, USA; danny-dhanasekaran@ouhsc.edu

**Keywords:** autophagy, lysosome, BAPX1, HDAC6, lysophosphatidic acid, epithelial to mesenchymal transition, ovarian cancer

## Abstract

Several soluble factors secreted by the stromal cells and cancer cells within the tumor microenvironment facilitate the progression and invasiveness of ovarian cancer. In ovarian cancer cells, lysophosphatidic acid (LPA) modulates the transcriptome profile and promotes cell invasiveness by the downregulation of autophagy. Here, we further elucidate this mechanism by focusing on the molecular and cellular events regulating autophagy. Transcriptomic and Western blotting analyses revealed NKX3-2, a transcriptional factor, to be among the genes hyperexpressed in LPA-stimulated ovarian cancer cells. Bioinformatic analyses revealed that in ovarian cancer patients, the expression of *NKX3-2* positively correlates with genes involved in cell motility and migration, while it negatively correlates with macromolecular catabolic pathways. In various ovarian cancer cell lines, NKX3-2 silencing abrogated LPA-induced cell migration. Mechanistically, this effect is linked to the restoration of the HDAC6-mediated relocation of the lysosomes in the para-golgian area, and this results in an increase in autolysosome formation and the overall upregulation of autophagy. Silencing the expression of *ATG7* or *BECN1*, two autophagy genes, rescued the migratory phenotype of the NKX3-2-silenced ovarian cancer cells. Taken together, these data reveal the mechanism by which the LPA-NKX3-2 axis promotes the invasiveness of ovarian cancer cells and supports the possibility of targeting NKX3-2 to reduce the migratory capacity of cancer cells in response to a permissive microenvironment.

## 1. Introduction

Ovarian cancer (OC) persists as a leading cause of mortality among women, accounting for the highest mortality rate compared to other gynecological malignancies [1]. The high heterogenicity of this malignancy represents a serious challenge toward a ‘one size fits all’ therapy. A precision medicine approach is desirable in the development of more effective therapeutic strategies for OC, and this requires the identification of novel molecular biomarkers serving as robust predictors of therapy response and of long-term outcomes [2].

The tumor microenvironment greatly influences OC progression by secreting soluble factors and metabolites that support the metabolic rewiring necessary for tumor growth and spread [3]. Lysophosphatidic acid (LPA) is a bioactive phospholipid found at high concentrations in the ascites of OC patients. Numerous studies have delineated the mitogenic and pro-tumorigenic role of LPA in OC biology, unveiling its ability to induce cancer cell migration and invasion, as well as to promote metabolic changes in both cancer cells and stromal cells (e.g., cancer-associated fibroblasts and macrophages) [4,5,6,7,8,9,10]. In ovarian cancer, LPA acts as an autocrine stimulator of cell proliferation and cell migration by interacting with LPA receptors, mainly via the G12 protein α subunit encoded by the oncogene *GNA12* [11]. Transcriptomic analysis of SKOV3 ovarian cancer cells treated with LPA indicated that GNA12 drives the decreased expression of ATG16L1, a protein involved in autophagosome formation [11].

Our group has recently shown that LPA promotes ovarian cancer cell migration by downregulating autophagy via Hedgehog-mediated signaling [12]. We therefore hypothesized that a thorough analysis of the transcripts regulated by LPA could reveal the genes involved in ovarian cancer cell migration and impacting on the autophagy pathway. One such gene that emerged from the transcriptomic analysis of LPA-treated SKOV3 cells is *NKX3-2* (NK3 homeobox 2), also known as BAPX1, a transcriptional repressor belonging to the NK family of homeobox-containing proteins. NKX3-2 was shown to promote the migration of gastric cancer cells [13]. However, there are no data in the literature about its role in the regulation of autophagy. Therefore, this study aimed to investigate whether and how NKX3-2 could mediate LPA effects on cell migration and autophagy in ovarian cancer cells. Starting from the evidence that LPA upregulates NKX3-2 in different OC cell lines, in the present work, we describe the pro-tumorigenic role of NKX3-2 in OC biology. More specifically, we show for the first time that NKX3-2 promotes LPA-induced OC cell migration and concomitantly inhibits autophagy by modulating lysosome transport via the downregulation of HDAC6. Lysosomal positioning has emerged as an important regulator of autophagy, and HDAC6 plays a role in this regulation by facilitating the autophagosome-lysosome fusion [14,15]. Accordingly, NKX3-2 knockdown results in autophagy induction by restoring the autolysosome formation at the microtubule-organizing center (MTOC) region.

Taken together, our data indicate that NKX3-2 may harbor a promising potential as an oncogenic factor, thus prompting future studies to deeper elucidate its biological significance and potential as an anti-cancer therapeutic target.

## 2. Materials and Methods

### 2.1. Cell Culture and Treatments

OVCAR3 (cod. HTB-161) and SKOV3 (cod. HTB-77) cell lines were purchased from the American Type Culture Collection (ATCC) (Manassas, VA, USA), while the OAW42 cell line was obtained from ECACC (cod. 85073102; European Collection of Authenticated Cell Cultures; Porton Down, Salisbury, UK). The cells were maintained under standard culture conditions (37 °C, 5% CO_2_). The ovarian cancer cells were cultured as previously detailed in [12]. HDFa (cod. PCS-201-012; ATCC), HFF-1 (cod. SCRC-1041; ATCC), and skin fibroblasts (kindly provided by Prof. E. Grossini’s lab, Università del Piemonte Orientale, Novara, Italy) were cultured in RPMI1640 medium supplemented with 10% FBS, 1% glutamine, and 1% penicillin-streptomycin solution.

Chloroquine (ClQ, cod. C6628; Sigma Aldrich, St. Louis, MO, USA) was dissolved in sterile water and used at a 30 µM final concentration. 1-Oleoyl Lysophosphatidic acid sodium salt (LPA, cod. 22556-62-3; Cayman Chemical, Ann Arbor, MI, USA) was dissolved in DMSO and used at a 20 µM final concentration.

### 2.2. Antibodies

The following primary antibodies were employed for either immunofluorescence or Western blotting: rabbit anti-LC3 (1:2000, cod. L7543; Sigma Aldrich), rabbit anti-p62/SQSTM1 (1:500, cod. 8025; Cell Signaling Technologies, Danvers, MA, USA), rabbit anti-HDAC6 (1:500 for WB, 1:250 for IF, cod. NBP1-61625; Novus Biologicals, Centennial, CO, USA), mouse anti-β-tubulin (1:1000, cod. T5201; Sigma Aldrich), rabbit anti-NKX3-2 (1:500, cod. PA5-21108; Invitrogen, Waltham, MA, USA), rabbit anti-GAPDH (1:1000, cod. G9545; Sigma Aldrich), mouse anti-LC3 (1:100, cod. 0260-100/LC3-2G6; Nanotools, Teningen, Germany), mouse anti-LAMP1 (1:1000, cod. 555798; BD Biosciences, Franklin Lakes, NJ, USA), rabbit anti-LAMP1 (1:200, cod. sc-5570; Santa Cruz Biotechnology, Santa Cruz, CA, USA), mouse anti-γ-tubulin (1:500, cod. T5326; Sigma Aldrich), rabbit anti-mTOR (1:500, cod. 2983; Cell Signaling Technologies), mouse anti-N-cadherin (1:50, cod. 610920; BD Biosciences), mouse anti-dynein (1:500, cod. MAB1618; Sigma Aldrich), mouse anti-BECLIN-1 (1:100, cod. 612112; BD Biosciences), and rabbit anti-ATG7 (1:500, cod. 04-1055; Millipore, Burlington, MA, USA). The secondary antibodies utilized for Western blotting were HRP-conjugated goat anti-mouse (cod. 170-6516) and goat anti-rabbit (cod.170-6515) (both diluted 1:10,000, BioRad, Hercules, CA, USA). The secondary antibodies used for immunofluorescence were AlexaFluor488-conjugated goat-anti-rabbit IgG antibody (cod. A32731) and AlexaFluor555-conjugated goat-anti-mouse IgG antibody (cod. A32727) (both diluted 1:1000, Thermo Fisher Scientific, Waltham, MA, USA).

### 2.3. Wound Healing Migration Assay

The cells were seeded in Petri dishes and cultured until confluence. The cell monolayer was then scratched using a sterile pipette tip, and the cell debris was washed out with PBS. To prevent autophagy induction, the culture media were renovated every 24 h. At each time-point indicated, photos of the wound were acquired by phase-contrast microscope (magnification 5×, Zeiss AXIOVERT 40CFL, Zeiss, Oberkochen, Germany). The migration rate was calculated with ImageJ software (v.1.48), taking into consideration the area free of cells as previously reported in [12].

### 2.4. Western Blotting

The cells were plated in Petri dishes and treated as indicated. Protein homogenates were prepared following the standard procedure. The samples were run on SDS-PAGE and then transferred on a PVDF membrane, as detailed in [12]. The filters were incubated overnight at 4 °C with a specific primary antibody solution, followed by incubation with a secondary HRP-conjugated antibody for 1 h at room temperature. The detection was performed using Enhanced Chemiluminescence reagents (cod. NEL105001EA; Perkin Elmer, Waltham, MA, USA), and the bands were detected at the VersaDOC Imaging System (BioRad, Hercules, CA, USA) using Quantity One software (v.4.5). For normalization, the filters were re-probed with β-Tubulin or GAPDH. Densitometric analysis of the bands was performed by using Quantity One software (v.4.5). The densitometric data are given in arbitrary units.

### 2.5. Immunofluorescence

The cells were plated on sterile coverslips at the appropriate density and treated accordingly. Then, the coverslips were stained as previously reported in [12]. The samples were stained overnight at 4 °C with specific primary antibodies, followed by incubation with dye-conjugated secondary antibodies and DAPI for 1 h at room temperature. The coverslips were mounted onto glasses using SlowFade reagent (cod. S36936; Life Technologies, Paisley, UK) and imaged under a fluorescence microscope (Leica Microsystems, Wetzlar, Germany; DMI6000).

### 2.6. Transfection

The cells were plated on coverslips or Petri dishes (depending on the experiment performed) and allowed to adhere for 24–36 h before starting the transfection. Post-transcriptional silencing was achieved by small interference RNA (siRNA) technology. The cells were transfected with 100 pmol siRNA by using Lipofectamine 3000 Reagent (cod. L3000-015, Life Technologies). The treatments were performed 36 h after the transfection and followed up to a maximum of 72 h. The samples were then processed for immunofluorescence and Western blotting as described above. The sequences of the siRNAs were as follows: siNKX3-2 CCAAGAAGGUGGCCGUAAAUU; siHDAC6 AGAGAACUGCGACGAUUAAUU; siRNA scramble AGGUAGUGUAAUCGCCUUGTT.

NKX3-2 overexpression was achieved by transient transfection using Lipofectamine 3000 Reagent, following the purchaser’s instructions. The cells were grown for a subsequent 72 h, and then the samples were collected and processed for Western blotting as described above.

### 2.7. Chromatin Immunoprecipitation (ChIP)

The cells were plated in Petri dishes and treated as indicated. Before collection, the cells were subjected to crosslinking with 37% paraformaldehyde for 15 min at room temperature. After a quenching step with 125 mM glycine, the cells were harvested with an IP buffer supplemented by a protease inhibitors cocktail as detailed in [16]. The cell lysates were cleared by centrifugation, and sheared chromatin was obtained by repeated cycles of sonication (5 rounds of 15x1-s pulses at 50% power output). The samples were precipitated with 2 µg of anti-NKX3-2 antibody (cod. PA5-21108; Invitrogen) and incubated in an ultrasonic water bath for 15 min at 4 °C. Mock IP was performed without adding the antibody. The samples were then transferred to new tubes containing pre-cleared protein A-agarose beads and incubated at 4 °C for 45 min on a rotating platform. The samples were centrifuged and washed, and 100 µL of 10% Chelex 100 resin (cod. 1421253; BioRad) was added to each tube to capture the DNA. The samples were boiled for 10 min, centrifuged, and the supernatants containing the eluted precipitated chromatin were collected in new clear tubes. The purified DNA was characterized by PCR. Primers were designed to anneal the sequence of the promoter region of *HDAC6*. The sequences of the primers used were as follows: F-HDAC6 GGGCGGAGTTTGAGAAAG; R-HDAC6 CGTTTCGCTAACCCTCTTC. PCR amplification was performed using Taq DNA polymerase recombinant (cod. 10342-020, Invitrogen). The PCR product (expected~132 bp) was then characterized by agarose gel electrophoresis.

### 2.8. Assessment of Lysosome Positioning by Living Cell Imaging

The cells were plated on sterile coverslips and treated as indicated. At the end of the treatment, the cells were stained with Lysosensor Green-189 dye (cod. L7535, Invitrogen) for 30 min. The coverslips were washed thrice in PBS, mounted, and imaged with the fluorescence microscope (DMI6000, Leica Microsystems, Wetzlar, Germany).

### 2.9. Transwell Migration Assay

The cells were plated in Petri dishes and transfected as described above. After 48 h, the cells were trypsinized and counted before re-seeding them in the transwell chamber (cod. 3422, Corning Incorporated Costar, New York NY, USA) as follows. 50,000 cells were plated in serum-free medium within the upper chamber, while the bottom chamber of the transwell was filled with complete medium. After 24 h of growth in the incubator, the filter of each transwell (capturing the migrated cells to the underside of the inserts) was collected, washed with PBS, fixed in ice-cold methanol, and stained with hematoxylin/eosin solution (H&E, cod. 05-M06002, Bio-Optica, Milan, Italy) as reported in [12]. The H&E staining was acquired with the bright-field microscope (Panoramic Midi; Sysmex, Norderstedt, Germany). The statistical analyses were performed as follows: For each picture acquired at a 20× magnification, five different rectangles of fixed area were designed using the appropriate software and positioned in different zones of the filter to represent the heterogeneity of the migration process. Then, the cells within the rectangles were counted with ImageJ (v.1.48) and the ratio of “number of cells/area” of the rectangle was used to perform a statistical analysis to estimate the migratory potential in the different experimental conditions.

### 2.10. Microarray Genome-Wide Gene Expression Analysis

The cells were plated on Petri dishes, allowed to grow, and treated with 20 µM LPA for 72 h. The medium and treatments were renovated every 24 h. The total RNA isolation and sample processing were performed as previously reported in [12]. For the background correction, the normal exponential method was used, and quantiles were used for between-array normalization. To identify the differentially expressed genes (DEGs) between the control and LPA-treated cells, the LIMMA package (https://www.bioconductor.org/packages/release/bioc/html/limma.html, accessed on 29 October 2024) was used. To estimate the t-statistics, the empirical Bayes method was used. The criteria set for differential expression were the following: a log base two-fold change (logFC) greater than +0.20 for upregulated genes, or lower than −0.20 for downregulated genes, respectively.

### 2.11. TCGA Analysis

The expression data were retrieved from the ovarian cystadenocarcinoma dataset of The Cancer Genome Atlas (TCGA) (https://www.cbioportal.org, accessed on 14 March 2024). The classification of the patients into low versus high groups was defined relative to the median mRNA expression level. Statistical analyses were performed by R (v.3.6.1; The R Foundation for Statistical Computing, Vienna, Austria) and SAS software (v.9.4; SAS Institute Inc., Cary, NC, USA).

### 2.12. Bioinformatic Analysis

The volcano plot representing the LPA-differentially expressed genes (DEGs) was obtained by using TBtools software (v.2.085) (https://github.com/CJ-Chen/TBtools/, accessed on 20 July 2024). The cut-off criteria for the identification of the DEGs were the following: a *p*-value of ≤ 0.01 (the *p*-value threshold was fixed above 2.0) and |log2 fold change| ≥ 1.

The Gene Ontology (GO) analysis representing the biological process enrichment correlated to *NKX3-2* expression was obtained by using DAVID software (v.6.8) (https://david.ncifcrf.gov/, accessed on 5 July 2024). The bar graphs report the number of transcripts belonging to each up- or downregulated pathway.

ISMARA—Integrated System for Motif Activity Response Analysis (https://ismara.unibas.ch, accessed on 15 March 2024) is a bioinformatic online tool that models genome-wide expression, or ChIP-seq data, in terms of the computationally predicted regulatory sites for transcription factors. In this study, the ISMARA tool was used to retrieve the predicted top targets of NKX3-2 and the Gene Ontology (GO) processes in which it has been predicted to be involved.

### 2.13. Imaging Acquisition and Analysis

The acquisition of the fluorescence images was performed by two independent investigators unaware of the experimental conditions. The fluorescence staining was repeated three times in independent experiments, and five to ten microscopic fields randomly chosen were imaged. The quantification of the fluorescence intensity was performed with the software ImageJ (v.1.48).

### 2.14. Statistics

GraphPad Prism software (v.6.0) was used to calculate statistical significance and to generate graphs. Depending on the univariate or multivariate analysis, one-way or two-way ANOVA were used to determine the statistical differences among the experimental groups. For all experiments, a two-tailed *p*-value less than 0.05 was considered statistically significant.

## 3. Results

### 3.1. LPA Upregulates the Transcription of NKX3-2 in Several Ovarian Cancer Cell Lines with Different Genetic Backgrounds

**Figure 1:**
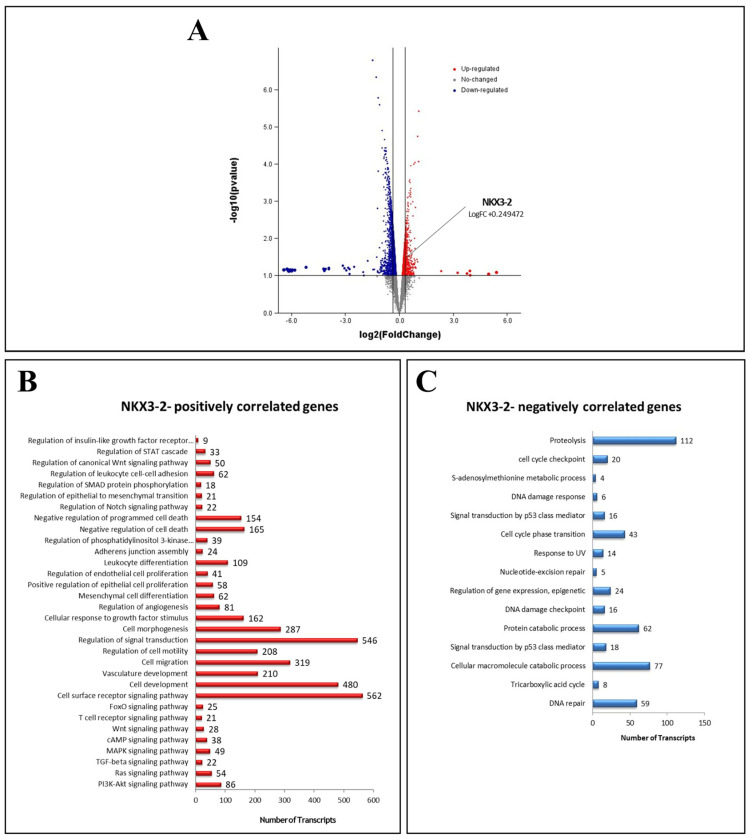
*NKX3-2* emerges among LPA-overexpressed transcripts involved in cell motility and downregulation of autophagy. (**A**) Volcano plot generated from transcriptomic analysis of SKOV3 ovarian cancer cells treated with 20 µM LPA for 72 h. Red dots represent upregulated genes with log2 (Fold Change) value > 1.0, while blue dots represent downregulated genes with log2 (Fold Change) value < −1.0. (**B**,**C**) Expression data were retrieved from TCGA ovarian cystadenocarcinoma dataset, and patients were stratified based on *NKX3-2* mRNA expression (high vs. low). Graphs report number of transcripts belonging to each biological process and pathway positively (**B**) or negatively (**C**) associated with *NKX3-2* mRNA expression.

First, we obtained the transcriptomic profile of SKOV3 cells treated with LPA for 72 h using an Agilent microarray platform [12]. Compared to the untreated cells, the treatment upregulated 321 transcripts and downregulated 1141 transcripts. The screening of the differentially expressed genes belonging to cell motility and invasive features shows the presence of *NKX3-2* among the top-significant LPA-upregulated transcripts (Figure 1A).

Next, we retrieved from TCGA the differentially modulated biological processes and pathways associated with *NKX3-2* expression. Among the processes with the highest number of transcripts modulated, we found that *NKX3-2* positively correlates with genes involved in cell motility and migration (Figure 1B), while it negatively correlates with transcripts regulating proteolysis and macromolecules catabolism (Figure 1C), prompting us to further investigate the involvement of NKX3-2 in these processes.

**Figure 2:**
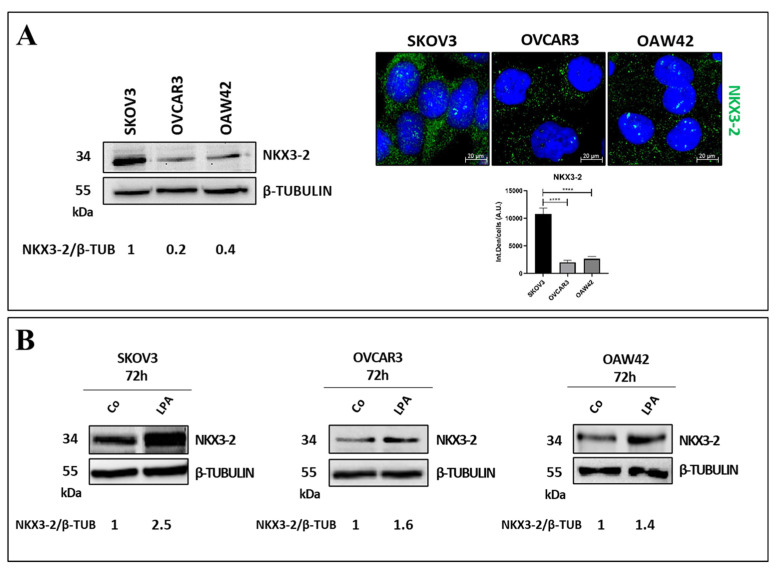
LPA upregulates NKX3-2 expression in different OC cell models. (**A**) Basal expression of NKX3-2 was evaluated by Western blotting, while its subcellular localization was monitored by immunofluorescence. Densitometric analysis of Western blotting, represented as NKX3-2/β-TUBULIN ratio, is included. Quantification of fluorescent signals was performed with ImageJ software. Histogram reports average ± S.D. One-way ANOVA test was performed. Significance was considered as follows: **** *p* < 0.0001. (**B**) SKOV3, OVCAR3, and OAW42 cells were treated with 20 µM LPA for 72 h. Western blot analyses were performed to assess modulation of NKX3-2 by LPA. Membranes were re-probed for β-TUBULIN to verify protein loading. Densitometric analysis of NKX3-2/β-TUBULIN ratio is included.

Then, we compared the basal expression of NKX3-2 in three different ovarian cancer cell lines, namely SKOV3, OVCAR3, and OAW42, that can be assumed as representative of the genotypic and phenotypic variety of ovarian cancers. As shown in Figure 2A, the SKOV3 cells exhibited the highest expression of NKX3-2 (about 80% higher than OVCAR3 and OAW42), which is localized in both the cytoplasm and the nucleus. Furthermore, we evaluated whether LPA also modulates NKX3-2 in other cell models. As shown in Figure 2B, the treatment with LPA for 72 h upregulated NKX3-2 expression in the OVCAR3 (1.6-fold) and the OAW42 cells (1.4-fold) as well, though to a lesser extent compared to SKOV3 (2.5-fold).

These results suggest that NKX3-2 is heterogeneously expressed in different OC cell lines, and it is part of the LPA-mediated downstream signaling regardless of its basal expression and the genetic background of the cancer cells.

### 3.2. NKX3-2 Knockdown Abrogates LPA-Induced Cancer Cell Migration

**Figure 3:**
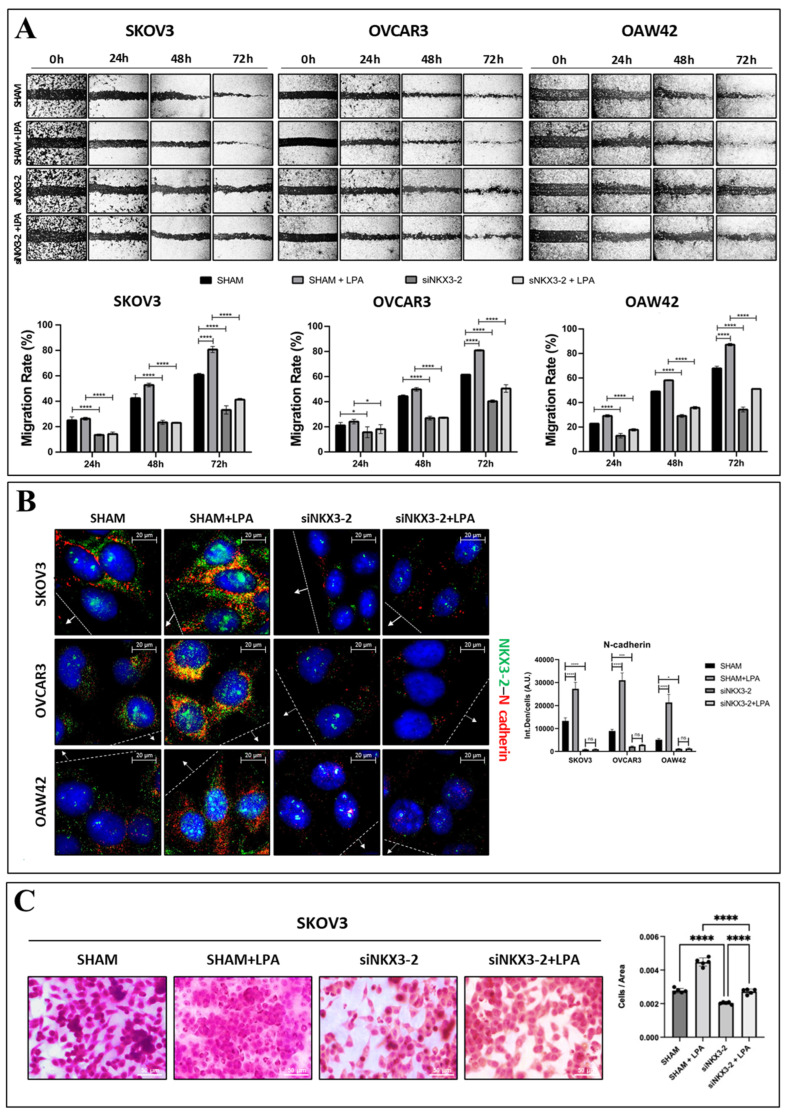
NKX3-2 supports cancer cell migration promoted by LPA. (**A**) SKOV3, OVCAR3, and OAW42 cells were plated on Petri dishes. Once grown to appropriate confluence, scratch across dish was performed using sterile pipette tip. Then, cells were transfected with siNKX3-2 or siRNA scramble. After 36 h from transfection, cells were either treated with 20 µM LPA or changed medium every 24 h. Phase-contrast photos were taken at 0, 24, 48, and 72 h. Histograms in bottom part report the rate of healing (%) for each experimental time-point estimated by ImageJ software. Data represent average ± S.D. calculated for three different fields per each condition. Two-way ANOVA test was performed. Statistical significance was considered as follows: **** *p* < 0.0001; * *p* < 0.05. (**B**) SKOV3, OVCAR3, and OAW42 cells were plated on sterile coverslips and treated as mentioned in panel (**A**). After 72 h from transfection, cells were fixed and stained for NKX3-2 (green)/N-cadherin (red). Quantification of fluorescence intensities was performed by ImageJ software. Histogram reports average ± S.D. calculated on three different fields for each condition in three separate experiments. Two-way ANOVA test was performed. Significance was considered as follows: **** *p* < 0.0001; *** *p* < 0.001; * *p* < 0.05. Scale bar = 20 µm; magnification = 63×. (**C**) Transwell assay was performed on SKOV3 cells. Cells were transfected and cultured (with or without LPA) for further 48 h. Then, cells were trypsinized counted, and 50,000 cells were re-plated within the upper chamber in serum-free medium and allowed to grow overnight. On following day (72 h time-point), cells were fixed and stained with H&E. Filters were mounted on glasses and acquired by bright-field microscope. Scale bar = 50 µm; magnification = 20×. Quantification of migrated cells was estimated by ImageJ software. Histogram represents average ± S.D. calculated on three different fields for each condition in three separate experiments. One-way ANOVA test was performed. Statistical significance was considered as follows: **** *p* < 0.0001.

We investigated the role of NKX3-2 in promoting LPA-induced OC cell migration. For this purpose, NKX3-2 was knocked down via post-transcriptional gene silencing in SKOV3, OVCAR3, and OAW42 cells. We performed a wound healing scratch assay to assess the cancer cell motility upon LPA challenge, and the healing rate was monitored through a time-course experiment. As shown in Figure 3A, NKX3-2-silenced cells exhibited more than a 30% decrease in their motility compared to that of their controls in all three cell lines. Notably, the knockdown of NKX3-2 (partially) abrogates the migration induced by LPA, as shown by the consistent reduction (about 30–40%) of the healing rate.

Next, we performed an immunofluorescence double-staining for NKX3-2/N-cadherin, focusing on the cells localized near the migration front (Figure 3B). We observed that the cells silenced for NKX3-2 exhibited a lower expression of N-cadherin compared to that detected in the controls. Moreover, the LPA-mediated upregulation of N-cadherin was greatly impaired in NKX3-2-silenced cells, thus corroborating the involvement of NKX3-2 in the LPA-induced epithelial-to-mesenchymal switch.

Additionally, as reported in Figure 3C, the transwell migration assay on SKOV3 cells shows that the silencing of NKX3-2 resulted in a consistent decrease in the number of cells migrated through the insert (about 25% and 35% less in the untreated and LPA-treated sham cells, respectively).

### 3.3. NKX3-2 Downregulates Autophagy in Ovarian Cancer Cells

Our bioinformatic data (shown in Figure 1C) identified proteolysis and macromolecules catabolism as the biological processes negatively correlated with *NKX3-2* expression. Thus, we decided to validate in vitro the functional relationship between NKX3-2 and autophagy.

**Figure 4:**
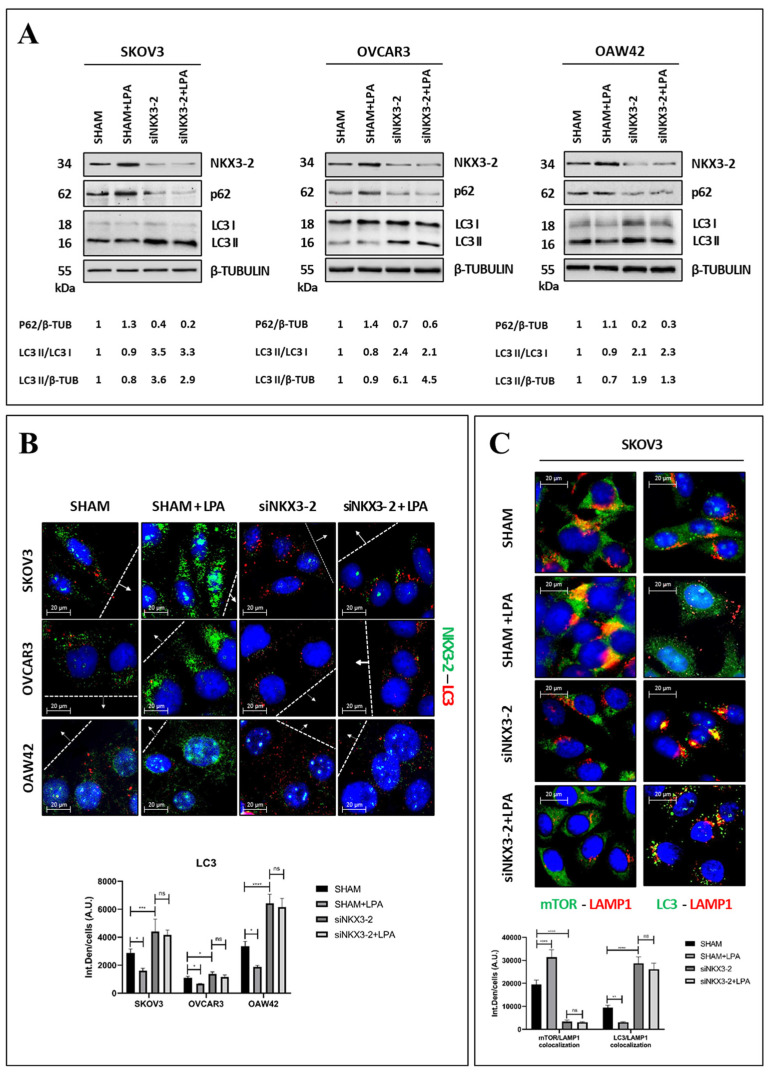
NKX3-2 knockdown restores LPA-induced inhibition of autophagy. (**A**) SKOV3, OVCAR3, and OAW42 cells were grown in Petri dishes and then transfected with siNKX3-2 or siRNA scramble. After 36 h from transfection, cells were either treated with 20 µM LPA or changed medium every 24 h. Cell homogenates were collected after 72 h and analyzed by Western blotting for expression of LC3 and p62. Efficiency of gene silencing was proved by assaying NKX3-2 levels. Filters were re-probed for β-TUBULIN to verify protein loading. Densitometric analysis of the blot is included. (**B**) SKOV3, OVCAR3, and OAW42 were plated on sterile coverslips, transfected, and cell monolayers were scratched with pipette tip. After 36 h from transfection, cells were either treated with 20 µM LPA or changed medium every 24 h. Coverslips were fixed after 72 h and stained for NKX3-2 (green)/LC3 (red). (**C**) SKOV3 cells were plated on coverslips, transfected, and cultured as described in panel (**A**). Cells were fixed and stained for (i) mTOR (green)/LAMP1 (red) and (ii) LC3 (green)/LAMP1 (red). Scale bar = 20 µm; magnification = 63×. Quantification of fluorescence intensities was performed using ImageJ software. Histograms report average ± S.D. calculated on three different fields for each condition in three separate experiments. Two-way ANOVA (panel (**B**)) and one-way ANOVA (panel (**C**)) tests were performed, respectively. Significance was considered as follows: **** *p* < 0.0001; *** *p* < 0.001; ** *p* < 0.01; * *p* < 0.05; ns *p* > 0.05.

Initially, we evaluated the autophagy modulation via Western blotting by monitoring the expression of two autophagy markers, LC3 and p62 (Figure 4A). We observed that in all of the three cell lines, the silencing of NKX3-2 resulted in autophagy induction demonstrated by the higher autophagosome production, indicated by the densitometric ratio of LC3-II over β-TUBULIN, and the faster autophagic flux, expressed as the densitometric ratio of LC3-II over LC3-I, together with an increased clearance of p62 compared to the trends observed in the controls. Notably, LPA treatment decreased the autophagosome production, autophagic flux, and p62 degradation only in the sham-transfected cells (not in the NKX3-2-silenced ones).

Since the net accumulation of LC3-II in the cells is the result of the rate of autophagosome synthesis and degradation, we inhibited autophagy by co-incubating SKOV3 cells with chloroquine. In this condition, the level of LC3-II reflects the amount of protein synthesized during the treatment [17]. From the Western blotting shown in Supplementary Figure S1, it is evident that in the NKX3-2-silenced cells, the conversion of LC3-I into LC3-II is promoted, even when these cells were treated with LPA.

To further characterize the contribution of NKX3-2 in the mechanism proposed, we performed an immunofluorescence double-staining of NKX3-2/LC3, focusing on the cells in proximity to the migration front (Figure 4B). The autophagy induction was evaluated based on the amount of LC3 puncta, which reflects the formation of mature autophagic vacuoles. Coherently with the Western blotting results reported in Figure 4A, the silencing of NKX3-2 resulted in an increase in LC3 puncta compared to the amount observed in the controls. On the other hand, the treatment with LPA inhibited the formation of autophagic vacuoles, as shown by the reduction in LC3-positive dots. Interestingly, this effect induced by LPA was abrogated in the NKX3-2-silenced cells, indicating that NKX3-2 may be a novel negative regulator of autophagy.

Next, we performed two immunofluorescence double-stainings for mTOR/LAMP1 (to monitor whether mTOR is active and inhibits autophagy) and LC3/LAMP1 (as meaning of the autolysosome formation) in SKOV3 cells. The results shown in Figure 4C confirmed NKX3-2 as an inhibitor of autophagy. Accordingly, the NKX3-2-silenced cells (either treated or not with LPA) exhibited a decreased lysosomal localization of mTOR in parallel with an increased LC3-LAMP1 co-localization, both indicative of autophagy induction.

We also investigated the role of NKX3-2 in the regulation of autophagy in normal cells. For this purpose, we selected the fibroblasts because of their abundance in the microenvironment surrounding the tumor mass. We assayed the level of NKX3-2 in three fibroblast cell lines (HFF-1, HDFa, and skin fibroblasts). NKX3-2 was found basally undetectable or barely detectable in the normal fibroblasts (Supplementary Figure S2). Interestingly, the transgenic overexpression of NKX3-2 resulted in the downregulation of autophagy in all of the three fibroblast lines (Supplementary Figure S2).

### 3.4. NKX3-2 Modulates Lysosomal Positioning

We hypothesized that NKX3-2 downregulates autophagy by impairing the fusion between autophagosomes and lysosomes. This cellular outcome can be physically achieved by altering the positioning of either the autophagosomes or lysosomes within the cytoplasm. More precisely, the ability to promote the transport of these organelles toward the perinuclear region, precisely at the microtubule-organizing center (MTOC), has been shown to favor the formation of autolysosomes [14,18,19].

The data shown in Figure 4B do not display any evident changes in the localization of the autophagosomes in the NKX3-2-silenced cells, thus suggesting that their positioning is not modulated by this protein. On the other hand, the immunofluorescence panels in Figure 4C showed that the siNKX3-2-transfected cells displayed a modest clustering of lysosomes towards the perinuclear region. The latter observation prompted us to more deeply investigate whether NKX3-2 could effectively modulate lysosomal positioning.

**Figure 5:**
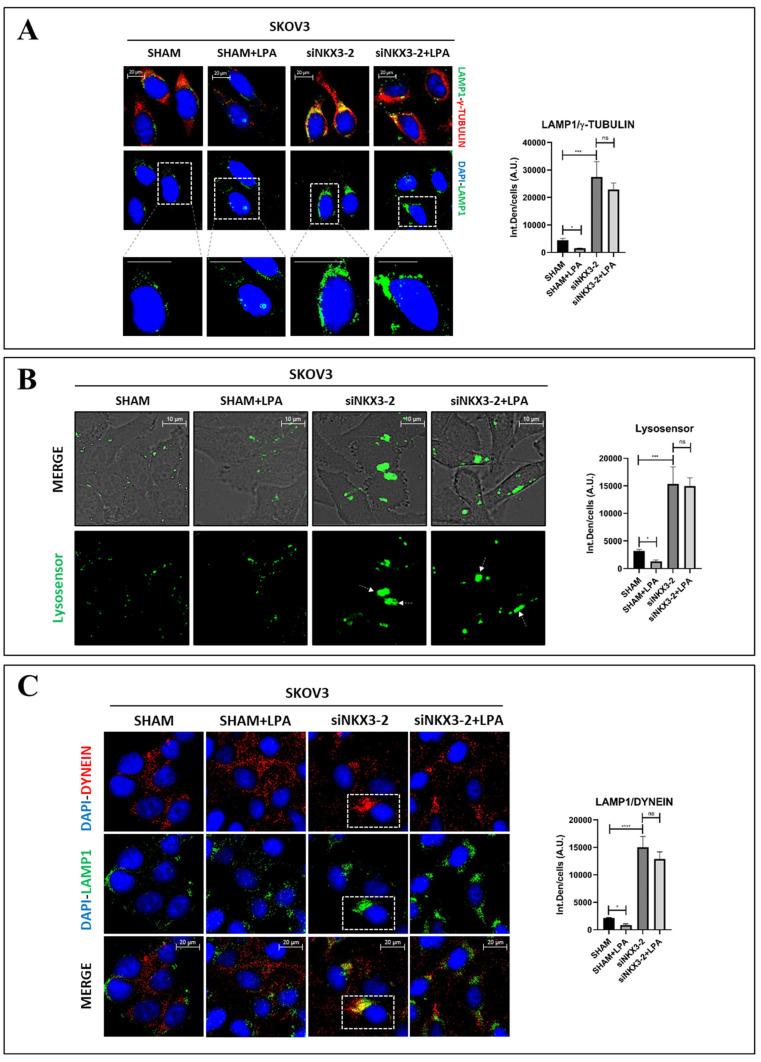
NKX3-2 regulates LPA-mediated lysosomal positioning. (**A**) SKOV3 cells were plated on sterile coverslips, then transfected with siNKX3-2 or siRNA scramble. After 36 h of transfection, cells were treated or not with 20 µM LPA, and media were changed every 24 h. Coverslips were fixed after 72 h and stained for LAMP1 (green)/γ-tubulin (red). The bottom panel of the figure provides zoom-in details of the lysosome positioning. Scale bar = 20 µm. (**B**) Cells were transfected and cultured as detailed in panel (**A**). At end of experiment, living cells were stained with Lysosensor dye for 30 min. Then, coverslips were mounted on glasses and immediately acquired by fluorescent microscope. (**C**) Cells were processed as described in panel (**A**), fixed, and stained for LAMP1 (red)/dynein (green). Scale bar = 20 µm; magnification = 63×. Quantifications of co-localization (panel (**A**,**C**)) and fluorescence intensities (panel **B**) were performed by using ImageJ software. Histograms report average ± S.D. calculated on three different fields for each condition in three separate experiments. One-way ANOVA test was performed. Significance was considered as follows: **** *p* < 0.0001; *** *p* < 0.001; * *p* < 0.05; ns *p* > 0.05.

To monitor the changes in the subcellular localization of lysosomes, SKOV3 cells were stained for LAMP1 and γ-tubulin, a constituent of the MTOC. As shown in Figure 5A, the NKX3-2-silenced cells displayed a marked clustering of lysosomes at the MTOC, as indicated by the co-localization of the two markers. Moreover, while LPA promoted the scattering of lysosomes across the cytoplasm in the sham-transfected cells, upon the same treatment, the lysosomes of the NKX3-2-silenced cells exhibited perinuclear localization.

To corroborate these findings, we performed a living cell imaging assay by labelling the SKOV3 cells with the Lysosensor dye, an acidotropic probe that accumulates in acidic organelles upon protonation. As can be seen in Figure 5B, the NKX3-2-silenced cells exhibited a marked perinuclear clustering of fluorescent signal. Additionally, we confirmed that the silencing of NKX3-2 abrogated LPA-mediated lysosomal scattering, resulting in a modest accumulation of lysosomes in proximity to the perinuclear region.

Next, we investigated whether NKX3-2 knockdown can alter the interaction of the lysosomes with motor proteins, which are ultimately responsible for the physical transport of these organelles along microtubules across the cell. SKOV3 cells were stained for LAMP1 and dynein, a motor protein transporting lysosomes toward the perinuclear region. As shown in Figure 5C, LPA treatment promoted the peripheral scattering of the lysosomes, while NKX3-2 silencing resulted in a marked co-localization of LAMP1 and dynein near the perinuclear region (indicating an ongoing retrograde transport of lysosomes), also in the presence of LPA.

### 3.5. NKX3-2 Modulates Lysosomal Positioning and Autophagy via the Downregulation of HDAC6

**Figure 6:**
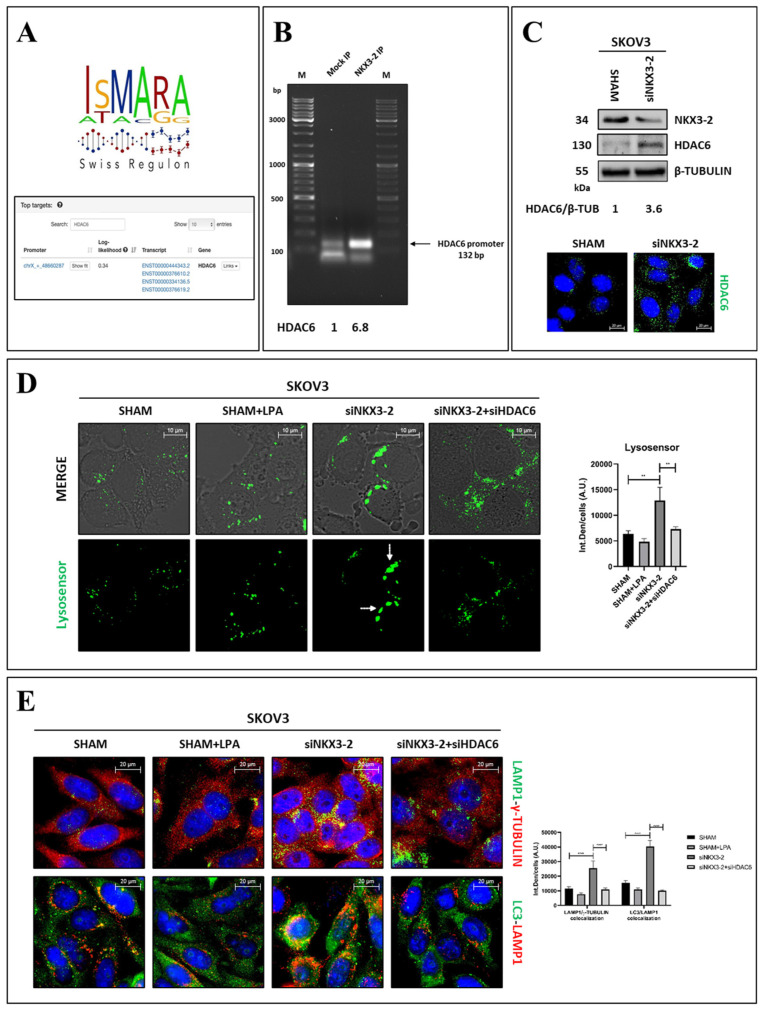
NKX3-2 modulates lysosomal positioning via HDAC6 downregulation. (**A**) ISMARA software was employed to identify predicted targets of NKX3-2 transcriptional activity based on its transcription binding site. Prediction identified *HDAC6* as target of NKX3-2. (**B**) In vitro validation of NKX3-2 binding on *HDAC6* promoter by chromatin immunoprecipitation in SKOV3 cells. Agarose gel electrophoresis shows enrichment of *HDAC6* PCR product in NKX3-2 immunoprecipitated (IP) fraction over Mock IP. Densitometric analysis is included. (**C**) SKOV3 cells were cultured in Petri dishes or on sterile coverslips. After 72 h from transfection with siNKX3-2 or siRNA scramble, cells were harvested and processed to evaluate HDAC6 expression by Western blotting and immunofluorescence. Densitometric analysis is included. (**D**) SKOV3 cells were initially transfected with siNKX3-2, and after 48 h, additional transfection with a mix of siNKX3-2 and siHDAC6 was performed. Then, cells were cultured for further 72 h, with media being renewed every 24 h. At end of experiment, living cells were stained with Lysosensor Green and subsequently imaged by fluorescence microscope. (**E**) Cells were processed as detailed in panel (**D**). Then, coverslips were fixed and stained for (i) LAMP1 (green)/γ-tubulin (red) and (ii) LC3 (green)/LAMP1 (red). Scale bar = 20 µm; magnification = 63×. Quantifications of fluorescence intensities panel (**D**) and co-localizations panel (**E**) were performed by ImageJ software. Histograms report average ± S.D. calculated on three different fields for each condition in three separate experiments. One-way ANOVA panel (**D**) and two-way ANOVA panel (**E**) tests were performed. Significance was considered as follows: **** *p* < 0.0001; ** *p* < 0.01.

We further dissected the molecular mechanism underlying NKX3-2-mediated lysosomal positioning. So far, NKX3-2 has been extensively studied for its activity as a transcriptional repressor. For this reason, we took advantage of the ISMARA software to predict, in silico, the putative targets of NKX3-2 transcriptional activity based on its transcription binding site. Among the many targets, we focused our search on proteins regulating the autophagy-lysosomal system, and we identified HDAC6 as a promising target candidate (Figure 6A). In addition to the deacetylase activity, HDAC6 has been studied for its ability to promote the fusion of autophagosomes and lysosomes by favoring their transport toward the MTOC [15,20,21,22], thus favoring autophagy induction. Therefore, we evaluated whether NKX3-2 may regulate lysosome transport via the modulation of HDAC6 expression.

Firstly, we validated in vitro the bioinformatic prediction by ChIP. Figure 6B shows the enrichment of *HDAC6* (about 6.8-fold) in the NKX3-2 immunoprecipitated fraction compared to the level observed in Mock IP, thus confirming *HDAC6* as a target of NKX3-2. Accordingly, the NKX3-2-silenced cells exhibited higher levels of HDAC6 compared to those of the control, as shown by the Western blotting and immunofluorescence analyses (Figure 6C).

Then, we addressed the potential involvement of HDAC6 in the regulation of lysosome transport upon NKX3-2 knockdown. As can be seen in Figure 6D, we monitored with Lysosensor dye how HDAC6 silencing affected the NKX3-2-regulated lysosome positioning. Interestingly, the co-transfection of siNKX3-2 + siHDAC6 resulted in the scattering of lysosomes across the cytoplasm, suggesting that HDAC6 may be the target through which NKX3-2 regulates the lysosomal retrograde transport.

Next, we investigated whether these observations could have functional consequences on autophagy. Again, we performed two double-staining immunofluorescences for LAMP1/γ-tubulin and LC3/LAMP1 (Figure 6E). Interestingly, the co-transfection of siHDAC6 + siNKX3-2 abrogated the phenotypes observed in the NKX3-2-silenced cells. In particular, the double-transfected cells exhibited scattered lysosomes, which did not co-localize with γ-tubulin, and a marked reduction in LC3-LAMP1 co-localization, indicative of the fusion between the autophagosomes and lysosomes.

### 3.6. NKX3-2 Promotes Cancer Cell Migration Through the Inhibition of Autophagy

**Figure 7:**
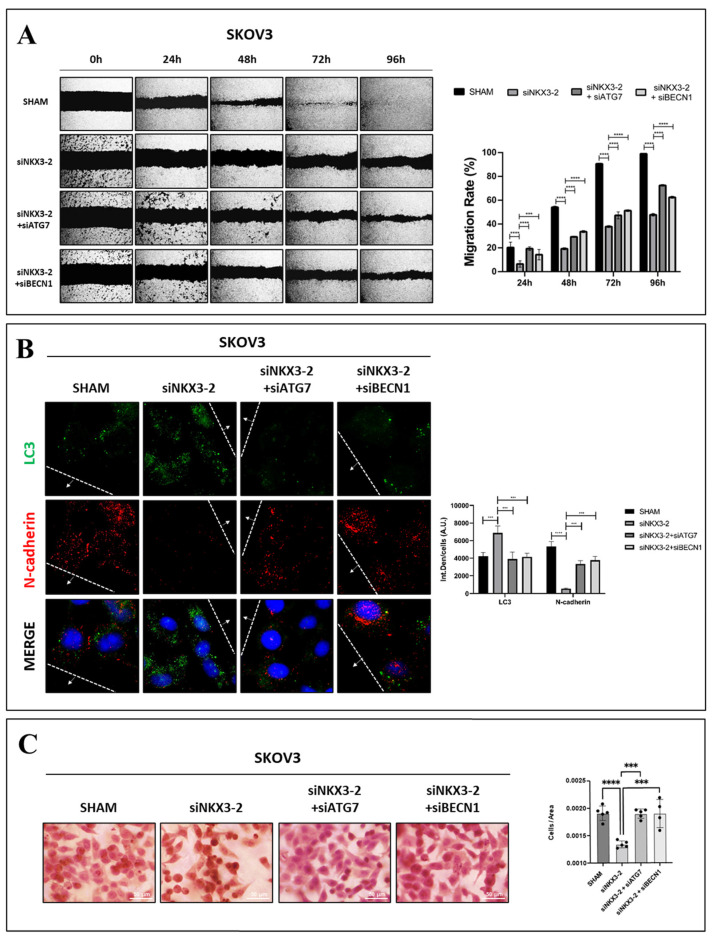
Knockdown of autophagy restores cancer cell migration in NKX3-2-silenced cells. (**A**) SKOV3 cells were initially transfected with siNKX3-2, and after 48 h, cells were trypsinized, counted, and re-seeded at same density. On following day, cell monolayers were scratched to produce a wound, and additional transfection with a mix of siNKX3-2 and siATG7 or siBECN1 was performed. Then, cells were cultured for further 96 h, with media being renewed every 24 h. Phase-contrast photos were taken at 0, 24, 48, 72, and 96 h. Histograms in bottom part report rate of healing (%) for each experimental time-point, estimated by ImageJ software. Data represent average ± S.D. calculated for three different fields per each condition. Two-way ANOVA test was performed. Statistical significance was considered as follows: **** *p* < 0.0001; *** *p* < 0.001. (**B**) SKOV3 cells were plated on sterile coverslips and were treated as previously described in panel (**A**). At end of experiment, cells were fixed and stained for LC3 (green)/N-cadherin (red). Scale bar = 20 µm; magnification = 63×. Quantification of fluorescence intensities was performed by ImageJ software. Histogram shows average ± SD calculated for three different fields for each condition in three separate experiments. Two-way ANOVA test was performed. Statistical significance was considered as follows: **** *p* < 0.0001; *** *p* < 0.001. (**C**) SKOV3 cells were initially transfected with siNKX3-2, and after 48 h, cells were trypsinized, counted, and re-seeded at same density. On following day, additional transfection with a mix of siNKX3-2 and siATG7 or siBECN1 was performed. After 48 h, cells were counted, and 50,000 cells were seeded within the upper chamber of transwell in serum-free medium and allowed to grow for 24 h. On following day, transwell inserts were fixed and stained with H&E. Quantification of migrated cells was performed by ImageJ software. Histogram represents average ± SD calculated for three different fields for each condition in three separate experiments. One-way ANOVA test was performed. Statistical significance was considered as follows: **** *p* < 0.0001; *** *p* < 0.001.

To conclude, we hypothesized that NKX3-2 promotes OC cell migration via the downregulation of autophagy. To test this, we evaluated how autophagy knockdown may affect NKX3-2-silenced cancer cell motility. The wound healing scratch assay shows that the knockdown of autophagy (either by siATG7 or siBECN1) rescues the migration of the NKX3-2-silenced cells, as demonstrated by the increased healing of the wound (about 25% and 20%, respectively) (Figure 7A). As a counterproof, the immunofluorescence staining of the cells in proximity to the migration front shows that the NKX3-2-silenced cells exhibit an increased expression of two autophagy proteins, namely BECLIN-1 and ATG7, supporting the view that autophagy is rescued in the absence of NKX3-2 (Supplementary Figure S3).

Furthermore, we performed an immunofluorescence double-staining for LC3/N-cadherin, focusing on the cells in proximity to the migration front. As shown in Figure 7B, the concomitant knockdown of NKX3-2 and of autophagy (either by siATG7 or siBECN1) resulted in a drastic reduction in LC3-positive spots in parallel with a marked upregulation of N-cadherin levels.

Finally, the transwell migration assay (reported in Figure 7C) shows that the additional transfection with either siATG7 or siBECN1 restored the migration capability of the NKX3-2-silenced cells; indeed, the number of migrated cells was like that observed in the control. Overall, these data confirm that the NKX3-2-mediated downregulation of autophagy is responsible for the enhanced migration of OC cells observed in vitro.

## 4. Discussion

The ovarian tumor microenvironment is considerably enriched in growth factors, cytokines, and other molecules capable of promoting tumor progression [3,23]. Among these, LPA is a mitogenic factor that has gained the attention of researchers due to its pleiotropic effects like promoting the glycolytic shift, which ultimately activates cancer-associated fibroblasts, the regulation of a pro-inflammatory cytokine network, supporting cancer cell motility and chemoresistance, and the differentiation of tumor-associated macrophages [5,7,8,10,12,24,25]. Thus, new therapeutic approaches may consider targeting the varied range of molecules mediating LPA-associated tumorigenic properties.

Autophagy is a conserved lysosomal-driven catabolic process aiming to ensure the correct turnover of old, or damaged, organelles and proteins [26]. Defective or deregulated autophagy can play a dual role in tumorigenesis depending on the stage of the disease [27]. On one side, in the early stages of tumorigenesis, autophagy prevents neoplastic transformations, preserves genomic stability, and opposes cancer cell migration and metastasis [28,29,30,31]. On the other hand, advanced-stage tumors exploit autophagy to survive and grow under stressful conditions, such as drug-induced genotoxicity and hypoxic and nutrient-depletion conditions [32,33].

We have recently shown that LPA stimulates ovarian cancer cell migration via the Hedgehog-mediated downregulation of autophagy [12]. Here, we further elucidated this mechanism by focusing on the molecular changes regulating autophagy. We have also reported that LPA downregulates the transcription of *ATG16L1* in ovarian cancer cells [11].

In this work, we found that NKX3-2 is upregulated by LPA in three OC cell models with different genetic backgrounds, stressing the generalization of such findings. Interestingly, the expression of NKX3-2 was found undetectable or barely detectable in normal fibroblasts. NKX3-2 belongs to a family of transcription repressors and appears to be involved in the regulation of cell migration and differentiation processes. NKX3-2 has been identified as a downstream effector of the Sonic hedgehog (Shh)/BMP and TGF-β-signaling pathways that promote epithelial-to-mesenchymal transition during embryonic development [13,34,35,36,37]. Additionally, NKX3-2 was shown to promote chondrogenic differentiation [38]. However, the mechanistic role of NKX3-2 in cancer biology has been poorly studied, and in few settings. NKX3-2 has been found hyperexpressed in different tumoral specimens compared to non-cancerous adjacent tissues [13,39,40,41,42]. A few studies reported the involvement of NKX3-2 in promoting the migration and invasion of gastric cancer cells [13] and the aberrant regulation of immune cell differentiation in B-cell lymphoma and T-cell acute lymphoblastic leukemia [39,40]. Additionally, NKX3-2 expression was positively correlated with tumor-associated macrophage infiltration in hepatocellular carcinoma, and this predicted a worse clinical outcome for those patients [42]. Furthermore, NKX3-2 was found significantly upregulated in chemoresistant ovarian tumors compared to chemosensitive ones, and it correlated with distant metastasis [43].

Our transcriptomic analyses performed in the TCGA ovarian cancer dataset revealed that *NKX3-2* expression positively correlates with genes involved in cell locomotion, while negatively correlating with transcripts belonging to the proteolysis and macromolecule catabolic processes. Thus, we investigated in vitro the role of NKX3-2 in OC cell migration and its relationship with autophagy.

The knockdown of NKX3-2 in three OC cell lines resulted in a marked reduction in cell motility, by means of the migration rate and expression of mesenchymal markers in the cells at the migration front. Notably, the silencing of NKX3-2 upregulated autophagy, thus pointing at this factor as a novel inhibitor of autophagy. Conversely, when overexpressed in normal fibroblasts, NKX3-2 inhibits autophagy. In cancer cells, NKX3-2 inhibits autophagy not only by negatively affecting late events, such as the fusion between autophagosomes and lysosomes, but also early events, such as mTOR recruitment to the lysosomal membrane and the autophagosome formation. In line with our previous findings [11], we hypothesize that under LPA stimulation, NKX3-2 could repress the transcription of *ATG16L1*.

Lysosomal positioning has recently emerged as a relevant subcellular event commonly affected and modulated during cancer progression [44]. Accordingly, the peripheral redistribution of lysosomes has been observed in advanced aggressive tumors, which exploit them to promote different cancer hallmarks, such as cell migration, extracellular matrix remodeling, and chemoresistance [45,46]. Consistent with its pro-tumorigenic role in ovarian cancer, LPA seemed to favor the peripheral scattering of the lysosomes, which was abrogated upon the silencing of NKX3-2.

Among the factors aiding motor proteins to mediate the retrograde transport of lysosomes, HDAC6 has been shown to associate with microtubules and orchestrate the retrograde transport of the lysosomes [15]. Our data demonstrate that NKX3-2 modulates the lysosomal positioning across the cell via the downregulation of HDAC6, eventually impairing the retrograde transport of the lysosomes toward the MTOC.

Lysosomal positioning has also emerged as a relevant process for autophagy regulation [14]. Here we demonstrated that NKX3-2 inhibits autophagy by hampering the retrograde transport of the lysosomes promoted by HDAC6, eventually decreasing their accumulation at the MTOC and thus impairing the formation of autolysosomes. These data provide further evidence supporting the pro-autophagic role of HDAC6 by means of favoring the lysosome transport towards the MTOC [15,22]. However, it remains still to be elucidated whether this mechanism relies on its deacetylase activity or the interaction with other factors, such as dynein motors.

Taken together, our data show that NKX3-2 promotes OC cell motility through the downregulation of autophagy, as the knockdown of the latter is able to partially restore the migration of cancer cells compared to the trend observed in the NKX3-2-silenced cells. Additionally, NKX3-2 silencing associates with the partial abrogation of LPA-induced cancer cell migration and the concomitant inhibition of autophagy, further consolidating the role of NKX3-2 as one of the main downstream effectors of the signaling cascade induced by LPA.

## 5. Conclusions

To our knowledge, our work represents the first report of the functional role of NKX3-2 in ovarian cancer by promoting cancer cell migration via the inhibition of autophagy. Mechanistically, NKX3-2 inhibits autophagy by impairing the retrograde transport of lysosomes via the downregulation of HDAC6. In conclusion, NKX3-2 may represent a novel oncogenic factor contributing to tumor progression. Our data may pave the way for future studies aimed at more deeply assessing its role in other cancer hallmarks, further providing a solid rationale to propose NKX3-2 as a novel therapeutic target in the tumors overexpressing NKX3-2.

## Data Availability

The clinical data retrieved for the in silico analysis are available in a publicly accessible repository (TCGA, Ovarian serous cystadenocarcinoma, TCGA Nature 2011) at [https://www.cbioportal.org/, accessed on 14 March 2024]. The experimental data presented in the study are included in the article/supplementary materials; further inquiries can be directed to the corresponding authors.

## Supplementary Materials

The following supporting information can be downloaded at https://www.mdpi.com/article/10.3390/cells13211816/s1: Supplementary Figure S1: SKOV3 cells were grown in Petri dishes, allowed to adhere, and transfected with siNKX3-2 or siRNA scramble. After 48 h from transfection, cells were incubated overnight (e.g., 16 h) with 30 µM chloroquine (ClQ). Cell homogenates were collected and analyzed by Western blotting for expression of LC3. Membranes were re-probed for GAPDH to verify protein loading. Densitometric analysis is included. Supplementary Figure S2: Fibroblast cell lines (HFF-1, HDFa, and skin fibroblasts) were transfected to overexpress NKX3-2. Cell homogenates were analyzed by Western blotting for expression of LC3. Membranes were re-probed for β-ACTIN to verify protein loading. Densitometric analysis is included. Densitometry is given in arbitrary units, assuming density of control (sham) bands as equal to one. Supplementary Figure S3: SKOV3 cells were seeded on coverslips, allowed to adhere, and upon reaching proper confluence were scratched and transfected with siNKX3-2 or siRNA scramble. Cells were fixed and stained for expression of ATG7 (green) and BECLIN-1 (red). Scale bar = 20 µm; magnification = 63×. Quantification of fluorescence intensities was performed using ImageJ software. Histograms report average ± S.D. *t*-test (two-tailed) was performed. Significance was considered as follows: * *p* < 0.05.
