# Supplementary material for: NKX3-2 Induces Ovarian Cancer Cell Migration by HDAC6-Mediated Repositioning of Lysosomes and Inhibition of Autophagy
Source: Cells. 2024 Nov 4;13(21):1816. doi: 10.3390/cells13211816 (PMC11544992; doi:10.3390/cells13211816)
Supplement: Supplementary file 1 [file cells-13-01816-s001.zip › cells-3205251-supplementary.pdf]

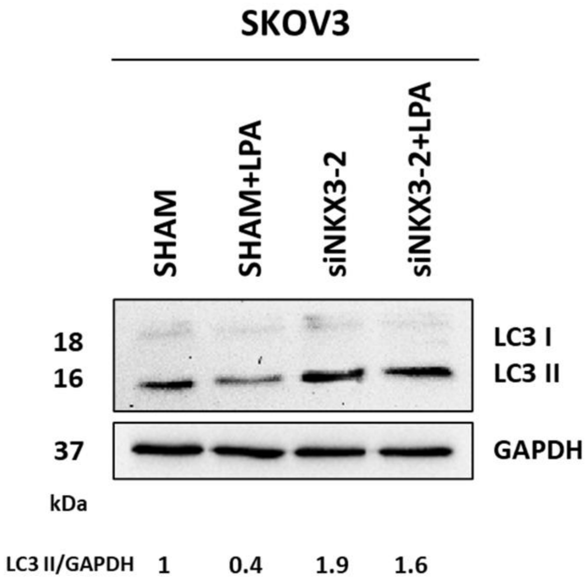

Supplementary Figure S1. SKOV3 cells were grown in Petri dishes, let adhere and transfected with siNKX3-2 or siRNA scramble. After 48 hours from the transfection, cells were incubated overnight (e.g. 16 hours) with 30 μM chloroquine (ClQ). Cell homogenates were collected and analyzed by Western blotting for the expression of LC3. Membranes were re-probed for GAPDH to verify protein loading. The densitometric analysis is included.

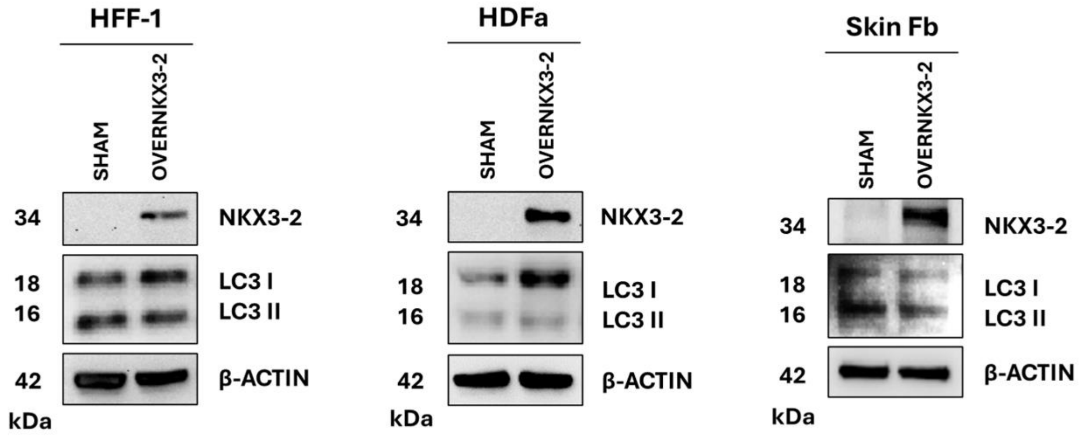

NKX3-2/β-ACT	1	7.3	NKX3-2/β-ACT	1	14.6	NKX3-2/β-ACT	1	20.1
LC3 II/LC3 I	1	0.6	LC3 II/LC3 I	1	0.4	LC3 II/LC3 I	1	0.8
LC3 II/β-ACT	1	0.8	LC3 II/β-ACT	1	1.1	LC3 II/β-ACT	1	0.6

Supplementary Figure S2. Fibroblast cell lines (HFF-1, HDFa, and primary skin fibroblasts) were transfected to overexpress NKX3-2. Cell homogenates were analyzed by Western blotting for the expression of LC3. Membranes were re-probed for β-ACTIN to verify protein loading. The densitometric analysis is included. Densitometry is given in arbitrary units, assuming equal to 1 the density of the control (sham) bands.

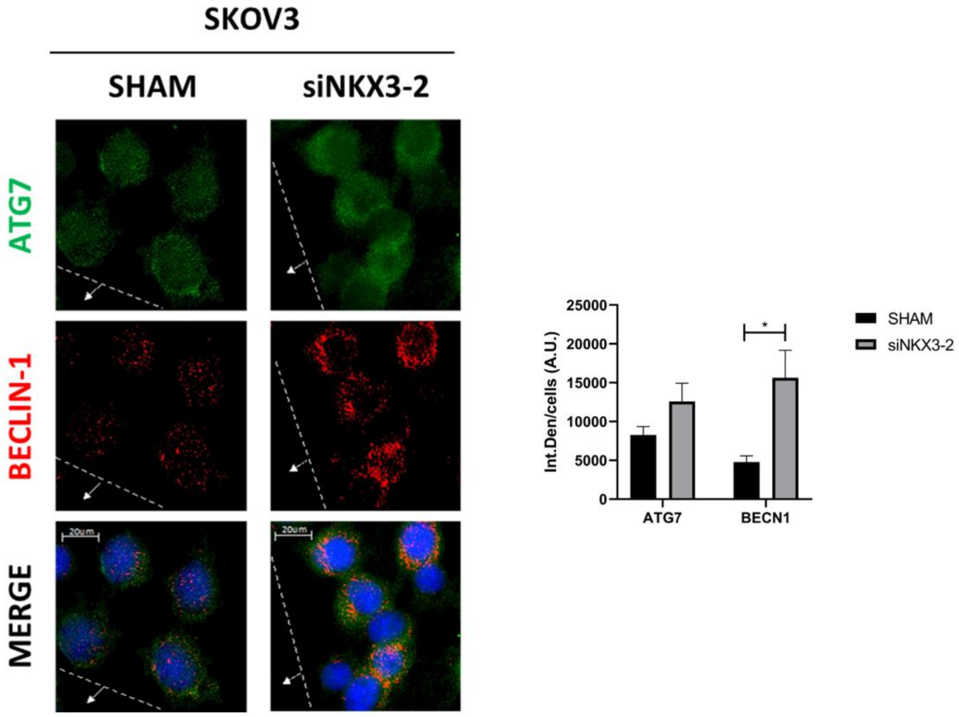

Supplementary Figure S3. SKOV3 cells were seeded on coverslips, let adhere and when reached the proper confluence were scratched and transfected with siNKX3-2 or siRNA scramble. Cells were fixed and stained for the expression of ATG7 (green) and BECLIN-1 (red). Scale bar = 20 µm; magnification = 63x. Quantification of fluorescence intensities was performed by using ImageJ software. The histograms report average ± S.D. T-test (two-tailed) was performed. Significance was considered as follows: * $p < 0.05$.
